# “Influencing the influencers:” a field experimental approach to promoting effective mental health communication on TikTok

**DOI:** 10.1038/s41598-024-56578-1

**Published:** 2024-03-11

**Authors:** Matt Motta, Yuning Liu, Amanda Yarnell

**Affiliations:** 1 Department of Health Law, Policy, & Management, Boston University School of Public Health, Boston, USA; 2 Center for Health Communication, Harvard University TH Chan School of Public Health, Boston, USA

**Keywords:** Health policy, Public health, Quality of life

## Abstract

A substantial body of social scientific research considers the negative mental health consequences of social media use on TikTok. Fewer, however, consider the potentially *positive impact* that mental health content creators (“influencers”) on TikTok can have to improve health outcomes; including the degree to which the platform exposes users to evidence-based mental health communication. Our novel, influencer-led approach remedies this shortcoming by attempting to change TikTok creator content-producing behavior via a large, within-subject field experiment (N = 105 creators with a reach of over 16.9 million viewers; N = 3465 unique videos). Our randomly-assigned field intervention exposed influencers on the platform to either (a) asynchronous digital (.pdf) toolkits, or (b) both toolkits *and* synchronous virtual training sessions that aimed to promote effective evidence-based mental health communication (relative to a control condition, exposed to neither intervention). We find that creators treated with our asynchronous toolkits—and, in some cases, those also attending synchronous training sessions—were significantly more likely to (i) feature evidence-based mental health content in their videos and (ii) generate video content related to mental health issues. Moderation analyses further reveal that these effects are not limited to only those creators with followings under 2 million users. Importantly, we also document large system-level effects of exposure to our interventions; such that TikTok videos featuring evidence-based content received over half a million additional views in the post-intervention period in the study’s treatment groups, while treatment group mental health content (in general) received over three million additional views. We conclude by discussing how simple, cost-effective, and influencer-led interventions like ours can be deployed at scale to influence mental health content on TikTok.

## Introduction

In recent years, social media platforms like TikTok have become an indispensable part of not only online networking but also mental health information seeking for both adolescents and adults^1,2^. On TikTok, videos tagged #mentalhealth have drawn nearly 44 billion views.

At the same time, though, some have suggested that the rise in social networking may be responsible (at least in part) for increases in poor adolescent mental health^3,4^. Past literature has directly connected the increase in adolescents’ mental health problems to exposure to social media use. For example, the increasing prevalence of anxiety, depression, self-harm, and suicide since 2010 were related to the emergence and surge of social media platforms^5,6^. Spending more time on social media is also associated with sleep deprivation^7^. And exposure to idealized images on social media was found related to body dissatisfaction and eating disorders^8^.

While previous research suggests that social media use, and use of the TikTok platform in particular, can have a negative impact on MH outcomes (see: ^9^ for a meta-analytic review), fewer have considered the possibility that the platform may also play a role in *improving* MH. Fewer still have explored the possibility of partnering with mental health content creators (MHCCs) to influence the supply of evidence-based mental health content on the platform. This is (perhaps) surprising, as there are many prominent content creators on the site—i.e., widely followed “influencers” on the platform—who produce videos that promote MH.

Importantly for public health, these MHCCs often reach audiences that are not utilizing the care they need—often due to structural inequities in access to mental health care—including teens or Black men. Many, like social worker and psychotherapist Nadia Addesi (@nadiaaddesi, TikTok following at time of study = 2.9 M) or psychiatrist Alok Kanojia (@healthygamer, TikTok following at time of study = 148.2 K), are licensed mental health providers who seek to broaden access to the mental health information they provide to patients. Others, like Samantha Chung (@simplifyingsam, TikTok following at time of study = 806.8 K) or Braden Kadlun Johnson (@kadlun, TikTok following at time of study = 138.7 K) use their lived mental health experience to decrease stigma and encourage people to seek support. And some, like psychiatrist Sasha Hamdani (@psychdoctormd, TiKTok following at time of study = 893.9 K) intentionally do both.

Whether or not MHCCs on TikTok can positively influence MH outcomes depends on (a) the availability of the types of MH communication thought to promote healthful attitudes and behavior, and (b) users’ exposure to it. At this point, users’ motivations and/or abilities to accept that evidence-based information and integrate it into memory then have the potential to alter the considerations they bring to mind when formulating opinions about (in this case) mental health^10^. Those updated opinions could, in turn, inspire behavioral change via one or both of the following mechanisms: (a) by leading individuals to experience an enhanced belief in their take action to improve their own mental health outcomes—thereby increasing the probability of doing so^11^, or (b) by increasing awareness of actions individuals can take to improve their health (a “priming” effect^12^).

Whether or not exposure to evidence-based mental health information on TikTok actually has these behavioral effects is an open question. The purpose of this study is to investigate a least one necessary preconditions under which these effects might occur; i.e., that it is possible to alter the supply of evidence-based mental health communication on TikTok.

Concerning the availability of mental health information, previous meta-analytic research finds that online-mediated exposure to mental health resources—such as mobile application interventions that provide information about clinical MH diagnoses and their potential treatments, and/or that provide training to participate in self-guided meditation, mindfulness exercises, and self-monitoring of healthy/unhealthy behavior—is associated with positive MH outcomes; including decreased self-reported anxiety and substance abuse^13,14^.

More generally, we might expect videos that promote *evidence-based MH communication* (EBMHC for short)—i.e., messages that contain information pertaining to the recognition and/or treatment of mental health issues that are informed by scholarly research—can have a positive influence on health attitudes and behaviors. For example, videos that talk about the mental health struggles associated with pregnancy and new motherhood might help social media users manage those feelings and encourage pregnant persons to seek mental health support during and before this period. Consistent with this view, previous research suggests that social media users’ exposure to videos promoting EBMHC is associated with improved mental health outcomes. For example, anti-stigma mental health interventions through video-based instruction in educational institutions have been shown to be successful in improving mental health literacy, attitudes, and beliefs towards mental health illnesses^15^.

Exposure to MH content on TikTok—evidence-based or not—can result from both incidental exposure and purposeful search activity. TikTok algorithms expose people who seek mental health information to related content. Unlike traditional media, which expose all readers/viewers to the same content, social media platforms apply complex and dynamic algorithms to personalize the content a user sees in their feed. These algorithms take users' past interactions on the platform and predict the content that is more likely to resonate with them, in order to keep the users engaged with the platform^16^. Therefore, when a user searches for mental health-related content on the platform, the algorithm finds related video content and distributes it to the user’s stream. Ultimately, this means that users can both passively encounter mental health content distributed by algorithm, or can actively curate their personalized information environment^17^.

The above review suggests that MH content is certainly present on the TikTok platform. The degree to which MH communication available on TikTok is *evidence-based*, however, remains an open question. Several studies offer anecdotal evidence of the prevalence of mental health misinformation on platforms like YouTube and Instagram^18–20^. But, efforts to do the same on TikTok are limited in their substantive scope^21^ and thereby represent an underdeveloped field of study.

The extent to which MHCCs might be persuaded to promote EBMHC on the platform is also an open area of research. To our knowledge, no previous studies have used rigorous randomized controlled trial (RCT) methods to collaborate directly with TikTok creators to promote EBMHC.

Ultimately, understanding the availability of EMBHC, and the dynamics by which MHCCs produce it, is a necessary first step in order to then assess whether interventions aimed at increasing exposure to evidence-based content might be associated with positive mental health outcomes.

Given previously-documented linkages between TikTok use and negative MH outcomes, we therefore devise a series of empirical tests aimed at assessing (RQ1) the degree to which MHCCs are already producing EBMHC, and (RQ2) whether or not researchers can intervene to *change* EBMHC behavior in order to increase the production of content that promotes positive MH outcomes.

We study the nature and prevalence of EBMHC on TikTok by attempting to influence MHCC behavior in the context of a field experiment. Specifically, we focus on creators’ references to what we term *Core Themes* from our training materials; i.e., references to a series of evidence-based talking points on a wide range of issues that pertain to mental health. Our research team developed the 5 Core Themes—as well as the EBMHC indicators we used to track them—via structured expert elicitation with mental health researchers and practitioners from across Harvard and its affiliated hospitals.

A detailed discussion of our EBMHC indicators and our field experimental protocol, can be found in the “Methods” section. Additionally, MO condition treatment materials documenting the scientific evidence underlying each of our Core Theme trainings, are provided as Supplementary Materials.

Briefly, after identifying a sampling frame of N = 105 prominent MHCCs on TikTok (see Table 2) we randomly assigned MHCCs to either receive an asynchronous digital toolkit outlining a series of evidence-based best practices for creating TikTok content that promotes MH outcomes (“Materials Only” or “MO”); receive both a toolkit *and* synchronous training at a month-long virtual summit held at Harvard’s TH Chan School of Public Health Center for Health Communication (“Conference plus Materials: CM”); or to receive no intervention at all. Whereas the MO condition can be thought about as a *passive* intervention aimed at promoting EBMHC content creation, the CM condition is an *active* attempt to do so.

We then content analyze pre-intervention videos produced by individuals included in our sampling frame to provide a sense of how much EBMHC content *already* existed on the TikTok platform, prior to our interventions (RQ1). After that, we compare change in pre- to post-intervention content production in order to assess the effectiveness of our interventions in changing content production behavior (RQ2); i.e., our ability to “influence the influencers.”

Note that the influencers involved in our study had a large “reach” (i.e., follower count) on the TikTok platform. Control group influencers totaled approximately 8.4 million followers at the onset of data collection, while those enrolled in the treatment groups totaled 8.5 million followers (cumulative reach = 16.9 million). Thus, we believe that changes in influencer content-producing behavior have the opportunity to reach many users on the TikTok platform.

## Results

### Core thematic analyses

#### Creator-level main effects

We begin our analysis by considering whether exposure to either of our two field experimental treatment conditions was associated with an increased likelihood that creators cited any of the core themes (our EBMHC indicators) listed in Table 1. To do this, we construct multilevel linear probability models (LPMs) that regress a dichotomous indicator of whether or not each video contained a reference to one or more of these topics on the interaction between treatment group assignment and a dichotomous pre/post intervention indicator; controlling for coder-level fixed effects, and random effects among content creators.

**Table 1:** Summary of core theme content indicators (EBMHC) included in pre/post-intervention content analysis.

EBMHC indicator	Example video & description
**Core Theme Reference: Mental Health Solutions that Scale** *Coder Instruction:* Does the video and/or video caption mention make mention of Core Theme #1: “Connecting People to the Help They Need?” Evidence that the video and/or caption made reference to this theme includes, but is not limited to words, phrases, and discussion of topics like… Mental health is a human right, yet access to care is limited Training community health workers to deliver mental health care is crucial Mental health apps hold promise in scaling care Investing in mental health not only improves the health of the population, but also stimulates economic development Detailed information about each topic is available at the following URL: https://osf.io/jn8m7/	In this TikTok, creator @LatinXTherapy discusses structural issues that limit access to care in her community URL: https://www.tiktok.com/@latinxtherapy/video/7239712810647817514
**Core Theme Reference: Maternal Health Matters** *Coder Instruction:* Does the video and/or video caption mention make mention of Core Theme #2: “How trauma spans generations?” Evidence that the video and/or caption made reference to this theme includes, but is not limited to words, phrases, and discussion of topics like… The impact of mental health disorders can span across generations Maternal depression and trauma can affect a child’s psychological development and mental health Mothers deserve more support throughout pregnancy and postpartum, and marginalized communities are disproportionately affected Detailed information about each topic is available at the following URL: https://osf.io/jn8m7/ *Valid Codes:* <1>NO <2>YES	In this TikTok, @DrBerryPsychologistReacts talks about how depression and anxiety can increase in pregnancy URL: https://www.tiktok.com/@drpatriceberry/video/7213002854758501678
**Core Theme Reference: Mental Health is Physical Health** *Coder Instruction:* Does the video and/or video caption mention make mention of Core Theme #3: “The Science Behind the Mind–Body Link?” Evidence that the video and/or caption made reference to this theme includes, but is not limited to words, phrases, and discussion of topics like… For mental health, prevention is crucial Poor mental health is directly connected to increase inflammation, cardiovascular disease, diabetes, and physical health problems Even in wealthy nations, just half of people with mental illness receive appropriate mental health care. But, not everyone who gets care gets quality care Detailed information about each topic is available at the following URL: https://osf.io/jn8m7/ *Valid Codes:* <1>NO <2>YES	In this TikTok, @Dr.KojoSarfo points to one way in which improving mental health can improve physical health: increased physical energy throughout the day URL: https://www.tiktok.com/@dr.kojosarfo/video/7233646161293282603
**Core Theme Reference: Discrimination Decays Your Mental Health** *Coder Instruction:* Does the video and/or video caption mention make mention of Core Theme #4: “The Corrosive Effect of Bias and Discrimination?” Evidence that the video and/or caption made reference to this theme includes, but is not limited to words, phrases, and discussion of topics like… People who encounter high levels of everyday discrimination have worse health outcomes compared to communities experiencing discrimination Structural racism produces a legacy of inequitable social and economic resources Appearance-based discrimination is a multi-billion dollar health crisis Detailed information about each topic is available at the following URL: https://osf.io/jn8m7/ *Valid Codes:* < 1>NO <2>YES	In this TikTok, creator @5hahem describes how weight stigma and body discrimination lead to a range of negative health effects, including psychological stress URL: https://www.tiktok.com/@dralfiee/video/7237310630624136494
**Core Theme Reference: Climate Action Must Include Mental Health** *Coder Instruction:* Does the video and/or video caption mention make mention of Core Theme #5: “The Truth Behind Climate Grief?” Evidence that the video and/or caption made reference to this theme includes, but is not limited to words, phrases, and discussion of topics like… Climate change exacerbates mental health issues Under-resourced, frontline communities are most affected by the detrimental effects of climate on mental health The mental health impacts of high temperatures and extreme weather must be incorporated into plans for the public health response to high temperatures Detailed information about each topic is available at the following URL: https://osf.io/jn8m7/ *Valid Codes:* <1>NO <2>YES	N/A. Although coders were provided with instructions regarding how to produce content in this area, we omitted this Core Theme from analysis due to low incidence (see: Data). We provide this information for the sake of transparency, only

Note that materials from the MO condition—which reference peer-reviewed evidence supporting each of our Core Thematic trainings—are available as Supplementary Materials.

The results are presented in full in Table S1, and summarized below. Note that inter-rater reliability estimates (Gwet’s AC) detecting the presence of each theme exceeded 0.90 in all cases. Please refer to the Extended Materials (Supplemental Table S2) for additional information.

**Figure 1:**
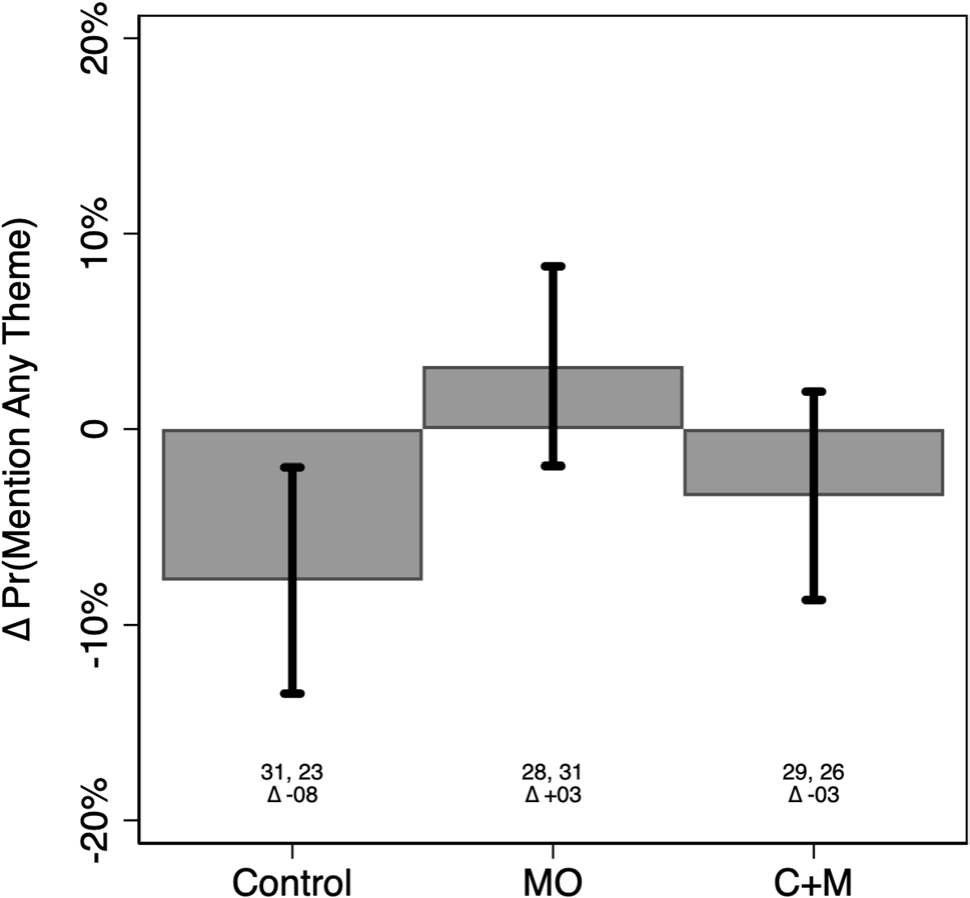
The Effect of Experimental Treatment Assignment on the Probability of Referencing Any of the Study’s Core Themes. Predicted probabilities (bars) hold all covariates at their sample means, and control for creator-level random effects. 95% confidence intervals extend out from each prediction, with model-adjusted based base rate probabilities listed below each bar.

Figure 1 summarizes the results presented in Table S1 by presenting contrastive marginal effects from the LPM described above. Bars correspond to pre/post-intervention change in the probability that creators reference any core theme in a video, with 95% confidence intervals extending out from each one. Predicted “base rate” probabilities (RQ1) from each stage of the field experimental procedure are listed below each bar, for reference (RQ2). These quantities can be interpreted as the relative effect of assignment to each of the study’s treatment (or control) conditions; controlling for assignment to all other conditions, and adjusting for coder fixed effects and creator-level random effects.

Table S1 reveals that just under one third of videos produced in the study’s Control Condition (pre-treatment) featured one of the study’s core themes. This serves as a baseline estimate of the prevalence of an important (albeit limited) dimension of EBMHC prevalence on TikTok (RQ1).

Encouragingly, though, exposure to both the MO (β = 0.10, p < 0.01) and CM conditions (β = 0.03, p = 0.62) is positively associated with an increased probability that creators include any of our recommended thematic content in their videos; relative to the control condition, and controlling for both fixed effect differences between coders and random effects among content creators. However, these findings only attain two-tailed significance at the p < 0.05 level in the MO condition.

Substantively, the predicted probabilities displayed in Fig. 1 suggest that pre/post-treatment movement in the MO condition is associated with a 3 percentage point increase in the likelihood that creators mention any of our core themes; from 28% (pre-intervention) to 31% (post-intervention). Although these predictions overlap slightly with the 0 line on the x-axis [p = 0.30; 95% CI: −0.03, 0.09], we emphasize that these estimates are derived from statistically significant parameter estimates described above, and reference pre/post-intervention change in predictions that are themselves significantly discernible from zero (Pre = 0.28, p < 0.01; Post = 0.31, p < 0.01).

Interestingly, the figure also shows that—although exposure to the CM condition was associated with a higher probability (26%) of referencing any of the study’s core themes than the control group (21%) in the post-treatment period—the relative change in content production (within the CM condition participants) actually decreased in the CM group over time. However, as these results are derived from parameter estimates that failed to attain statistical significance (and are not statistically discernible from zero, in the figure), we urge caution when interpreting these results, and instead focus our conclusions based on the results obtained from the MO condition.

The results presented thus far suggest that providing content creators with asynchronous, online training materials (MO) can have a powerful impact on mental health content production on TikTok. We also find little evidence that *adding* synchronous training sessions to these materials (CM) has a statistically appreciable influence on content production.

Still, some might ask if the relative inefficacy of the CM intervention may result from studying thematic references as a single, dichotomized outcome. In theory, it could instead be the case that exposure to *specific* training sessions—which were each devoted to particular themes—is associated with increased thematic content production in that particular content area, but not others.

We therefore assess whether creators who chose to attend each conference meeting were more likely to make references to the content covered in that session. We do this by modifying the results presented in Table S1, Column 1 to regress a dichotomous indicator (and their corresponding interactive terms; as described above) of whether each video referenced each core theme on an indicator of whether respondents were assigned to the MO condition, or the CM condition; with CM assignment partitioned by attendance at that session.

The results are presented in Table S2, which is otherwise structured analogously to Table S1. Note that, due to relatively low incidence (N = 1), we omit Core Theme #5 from these analyses.

The results again present something of a “mixed bag,” regarding the efficacy of the CM condition. Attendance at the workshops bore no statistically significant association with increased references to Core Themes 1 (β = −0.02, p = 0.66), 3 (β = −0.09, p = 0.21) and 4 (β = 0.00, p = 0.80). However, and in line with our a priori expectations, attendance at the workshop devoted to Core Theme 2 was positively (β = 0.07) associated with increased thematic content production, and approached conventional levels of two-tailed significance at p = 0.08.

Taken together, these results suggest that providing creators with asynchronous training materials (MO) is effective at changing creators’ content production habits on TikTok. Although these effects are relatively small in substantive size, their reach—given the size of the TikTok platform and potential audience for the types of content we study (see: System-Level Main Effects)—may be comparatively larger.

Further, we note that additional synchronous training workshops (CM) can, but do not always , also increase content production. Here, it is important to point out that failing to observe unique effects in the CM condition, above and beyond those documented in the MO condition, does not necessarily cast doubt on the usefulness of interventions with synchronous conference components. Rather, we think that this finding of results speaks to the comparative efficacy of the digital toolkits (present in both interventions) on MHCC behavior. Future research may benefit from including an additional treatment arm (“Conference Only”) that receives no digital materials in order to more-fully untangle this pattern of effects. Collectively, these findings have important implications for the scalability of our approach, as the dissemination of content creation guides (as we provide in the MO condition) is far less resource intensive than hosting live training sessions. Correspondingly, the comparative efficacy of MO condition suggests that a *simple and potentially-scalable interventional strategy* could have a significant impact on content creator behavior.

#### System-level main effects

Having documented the efficacy of our interventional approach—and the use of asynchronous training packets in particular—on content creator behavior, it is then worthwhile to consider the overall effects of deploying our interventions as a “system level” phenomenon. To do this, we aggregate video-level viewership meta-data, conducted in the month prior to and following the dissemination of our interventions (thereby standardizing temporal comparison across groups), among those content creators exposed to our study’s experimental interventions.

In so doing, we find that assignment to either of the study’s two experimental conditions *considerably altered mental health content on the TikTok platform*.

We find that videos referencing our Core Themes (see Fig. 1), produced by creators assigned to either of our treatment conditions, received 1,850,608 views post-treatment; relative to just 1,040,349 in the pre-intervention period for that group. This means that, irrespective of the effects that exposure to each treatment had on the content production behavior of individual creators, our field experiment appreciably (by over half a million views) increased the availability and viewership of evidence-based mental health content on TikTok.

### Analyses of mental health content production

Next, we recognize that even mental health content creators may use their personal social networking accounts on TikTok to post about a wide range of content both related *and unrelated* to mental health. Correspondingly, our interventional materials might not only encourage creators to produce content in line with the suggestions outlined in our training sessions and asynchronous materials, but to produce more mental health content altogether.

We test this possibility by constructing models analogous to those presented in Table S1, Column 1; swapping the dichotomous core theme reference indicator with a dichotomous indicator of whether or not coders determined that each video pertained to mental health (Gwett’s AC = 0.69). Additionally, these analyses necessarily include videos both related and unrelated to mental health when estimating the models. These results are presented in full in Table S1, Column 2.

As was the case in our thematic analyses, the main effects of our interventions across the full sample of content creators are associated with increases in the proportion of videos that pertain to mental health in only the MO condition (β = 0.06), which approach conventional levels of two-tailed significance at p = 0.06. Substantively, exposure to the MO condition is associated with a 5 percentage point increase in the likelihood of producing videos related to mental health; from 60% (pre-treatment) to 65% (post-treatment).

Importantly, system-level descriptive analyses (like those described in the preceding section) suggest that our interventions increased the visibility of mental health content on TikTok. Creators assigned to any of the study’s treatment conditions produced videos that earned over 6,399,733 views in the post-intervention period, relative to just 3,339,533 in videos produced beforehand (an increase in over 3 million views).

### Moderation by follower count

One potential objection that some might raise in response to the results presented thus far is that they may be confined to those content creators with the smallest “reach” (i.e., follower bases) on TikTok. In particular, some might argue that creators with *smaller* followings may be more receptive to making changes to the content they produce, in order to gain larger followings. This could imply that the effects of our treatment reach a sub-optimal audience size.

On the other hand, though, it could be the case that creators with *larger* followings feel as if they have a *greater* capacity to make changes to the content they produce, as these creators have already attracted large viewership bases on the site.

We test the possibility that the size of creators’ subscriber lists may influence their content-producing behavior by amending the models presented in the above section to interact experimental stimulus exposure with a dichotomous indicator of whether or not creators have over (46% of videos in our sample; 28% of creators sampled) or under (54%) 2 million followers on TikTok. Note that, as our study’s sampling frame is limited to just those creators with larger followings (see: Methods), the use of the terms “larger” vs. “smaller” applies specifically to the population under investigation, and not all TikTok content creators more generally.

In Table S4, we find that exposure to the MO (β = 0.10, p = 0.04), but not the CM (β = −0.05, p = 0.48) conditions is moderated by TikTok following size; such that we observe increased thematic content production among those with comparatively *larger* TikTok followings.

Probing only the statistically significant interaction term from these models, we find that MO exposure is associated with a 2 percentage point increase in the likelihood that those with large followings reference any of the study’s core themes; from 42% pre-treatment to 44% post-treatment. Interestingly, we document even stronger levels of change (an increase of 4 percentage points; from 24 to 28%) for creators with small followings, but at much lower baseline probabilities of referencing these topics.

Likewise, we again assess whether or not mental health content production *overall* might be influenced by creators’ follower counts. Base rate descriptive analyses suggest that creators with large followings were more likely to use their platforms to post about content unrelated to mental health (with 62% of videos pertaining to mental health), compared to those with relatively smaller followings (with 72% of videos pertaining to mental health). Correspondingly, we might expect to observe greater capacity for increased mental health content creation among creators with comparatively larger followings.

The results (see Table S4) suggest that creators with large followings who were assigned to the study’s MO condition (β = 0.16, p < 0.01), but not those in the CM group (β = 0.04, p = 0.53) were significantly more likely to produce videos related to mental health (although we note that the effects are positive in both cases). Extracting predictions only the statistically significant interaction term from the LPM, we find that assignment to the MO condition was associated with a 16 percentage point increase in the probability that a video pertained to mental health topics for creators with large followings; from 63% pre-treatment, to 79% post-treatment.

Collectively, these moderation analyses imply that the results observed above are *not* confined in their reach to only those content creators with the smallest followings on the platform.

## Conclusion

Our field experimental interventions aimed to increase the degree to which mental health "influencers" on TikTok incorporate evidence-based content into the videos that they produce. Collectively, the results presented throughout this manuscript suggest that content creators who were provided with simple, asynchronous training material toolkits were more likely to incorporate evidence-based mental health content into the videos that they produced (relative to a control group). Adding a synchronous conference component to the toolkit intervention did not result in a statistically significant increase in EBMHC production. We also note that the results presented throughout this manuscript are not confined to mental health influencers with comparatively large followings. More generally, our interventions increased viewership of mental health content on TikTok by over 3 million views throughout the study period; including over 800,000 additional views on videos that include evidence-based mental health content.

## Discussion

We recognize, of course, that this study is not without limitations. For example, although we attempted to recruit both an influential and diverse pool of creators to enroll in this study (please refer to the enrollment protocols listed in the Extended Materials), we nevertheless recognize that our sample represents an incomplete collection of content creators on TikTok. We further recognize that our randomization approach (please see Fig. 1 in Methods) may lead us to over-represent creators who are especially motivated to incorporate EBMHC into their work; which may potentially over-state treatment effects. We therefore look forward to future efforts to replicate studies like this one in more-heterogeneous samples of MHCCs.

Moreover, our field experimental treatments represent just one of *many* different asynchronous and/or synchronous interventions that we could—in theory—devise to influence creator behavior. Correspondingly, our interventions are necessarily limited in scope. This is true both with respect to the message properties that we study, as well as their substantive content (e.g., the five "core themes" that we assess in this study represent just some of many that we could potentially assess).

Finally, we note that our study takes place on just one social networking platform (TikTok) where content creators engage in mental health communication. We look forward to future efforts to extend this study's methods to other social networking platforms.

With that in mind, we note that—while our findings are necessarily limited—we believe that they nevertheless offer a scalable blueprint for future interventional research in this area. That is, because we document strong effects of asynchronous content creation material exposure on creators’ content-generation behavior, we believe that the methods we employ can be readily exported both to other social media platforms, and in service of inspiring evidence-based content generation in other areas; both related to mental health, and in other popular content domains as well.

More generally, we recognize that our work unpacks just one mechanism by which TikTok might improve MH outcomes (i.e., the availability of EBMHC). However, we see this work as a critical first step in harnessing TikTok’s potential to positively influence MH outcomes. In other words, demonstrating that this content *can or does* exist on the platform provides an empirical basis for researchers to then study how controlled exposure (e.g., in the context of a Randomized Controlled Trial; RCT) to TikTok-mediated EBMHC content can impact MH outcomes. We look forward to efforts to test this possibility in future research.

## Methods

### Study overview

The primary goal of our study is to determine if the video content produced by MHCCs on TikTok that were *treated* with our study’s interventions—being provided with an asynchronous online content creation toolkit (“Materials Only” Condition, or “MO”), or adding synchronous virtual summit component to the asynchronous materials (“Conference Plus Materials” Condition, or “CM”)—came to be more likely to produce EBMHC content one month following our intervention (see: Interventions) relative to one month before it, and in comparison to an untreated control group.

We focus specifically in this study on references to what we refer to as *Core Themes* discussed in our training materials. Core Thematic references include suggested talking points regarding how to convey the best available scientific evidence on a wide range of subjects that pertain to mental health; developed by the study team in consultation with the academic literature. A full list of Core Thematic content elements that we included in our coding scheme—including a rationale (Column 1) regarding why we believe that each one is demonstrative of EBMHC, and an example of this type of content on the platform (Column 2)—can be found in Table 1 in the Extended Materials.

The unit of analysis in this study is therefore not the N = 105 *influencers* we enrolled in the study (see: Recruitment), but the N = 3465 videos they created during the study period.

We study the effectiveness of our interventions by constructing a series of mixed effect models that regress several indicators of EBMHC content generation (e.g., efforts to appeal to the idea that mental health can impact one’s physical health; see Table 1) on indicators of whether or not each video was produced by influencers that were assigned to the study’s treatment vs. control groups, a time-varying pre/post-intervention indicator, and a term capturing interaction between the two. These models are structured as hierarchical linear probability models (LPMs), with influencer-level random effect variance components that account for the possibility of asymmetries in how influencers respond to our experimental treatments.

### Recruitment procedures: overview

**Figure 2:**
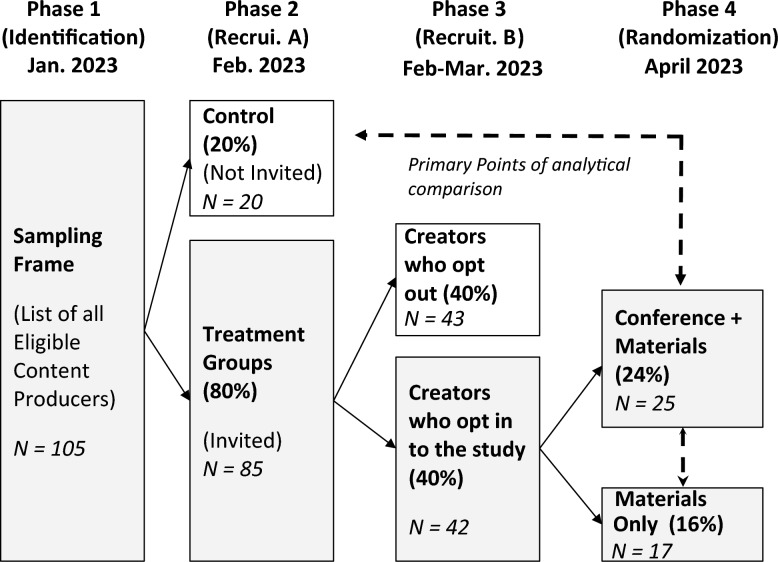
Visualization of Subject Recruitment & Treatment Assignment Procedures. Shaded bars reflect content creators who were deemed eligible (Phase 1) for inclusion in the study, and who then selected (Phase 2) and/or self-selected (Phase 3) into the final treatment randomization protocol (Phase 4). For reference, dashed arrows denote the primary points of analytical comparison in our study; i.e., each of the study’s two treatment groups (Phase 4), vs. both one another and the control group identified in Phase 2. All 85 respondents assigned to the study’s treatment group as eligible for Phase 2 were first contacted to participate in the study on 2/9/23, and those who did not apply were recontacted again on 2/21/23 and 2/28/23. Subjects could then determine whether to opt-in or opt-out of the study over the course of the next four weeks (noting that we deemed non-response to the recruitment emails as opting out of the study). For additional information about study protocols, please see Methods for a copy of the recruitment emails sent out in Phase 2. Please also visit the following Open Science Framework (OSF) Web Page https://osf.io/jn8m7/ for copies of all Phase 4 treatment materials. Finally, note that we carried out all Phase 2 and Phase 4 randomization procedures using the random integer generation command runiformint in Stata 15.

Recruitment for this study took place in three stages, which are summarized graphically in Fig. 2.

First, in Phase 1, we identified a sampling frame (i.e., list of social media accounts eligible for inclusion in our study) of N = 105 *mental health influencers* on TikTok. Our sampling frame includes individuals aged 18 or over who produce English-language mental health content, have at least 10,000 followers across TikTok or Instagram social media platforms, posted videos on the platform at least 4 times per month from December 2022—February 2023, and have been active on the site since February 2022. Please see the following section (Recruitment: Phase 1) for a detailed overview of how we both identified and selected accounts for inclusion into the sampling frame.

Next, in Phase 2a, we randomized all accounts in the sampling frame into study-wide treatment and control groups. To ensure high levels of enrollment in the study’s treatments, we assigned each influencer a 1 in 5 chance of being included in the study’s control group (N = 20), vs. a 4 in 5 chance of being included in the study-wide treatment (N = 85).

Influencers in the study-wide treatment group were then invited in Phase 2b to opt-in to the study’s experimental protocol, via participation in the mental health content creator summit described above (see: Intervention Design). Again, please consult the Extended Materials for additional information about the invitation procedure, including drafts of all recruitment materials.

Finally, in Phase 3, we further randomized all N = 42 influencers who opted-in to the study’s experimental protocol (Phase 2b) into two treatment groups. The “Conference + Materials” (CM) group was invited both to participate in our virtual mental health summit and receive a digital content creation training toolkit (described below), whereas the “Materials Only” (MO) group received only the latter. To ensure high levels of attendance at the study’s summit, we over-assigned participants into the CM group, such those who opted-in had a 3 in 5 chance of being selected into that group (vs. the MO group). This procedure left us with N = 25 influencers who attended the conference (CM), and 17 who were provided just with our digital toolkit.

This study was deemed exempt from institutional review by Harvard University’s Committee on the Use of Human Subjects, as it does not involve the analysis of human subjects data, and the need for informed consent was waived by the Institutional Review Board. All procedures were carried out in accordance with relevant guidelines and regulations.

### Recruitment phase #1: identifying mental health content creator “influencer” accounts

The first stage of our recruitment protocol involved identifying a universe (or “sampling frame”) of “influencer” accounts on the social networking site TikTok that would be appropriate for inclusion in this study. Specifically, we aimed to create a list of all English-language TikTok accounts, aged 18 or older who: (a) have a sufficiently large reach (as measured through social media “followers”), (b) post frequently on the platform about (c) topics related to mental health, and (d) have been posting on the platform for at least one year prior to the study period.

A full list of the specific criteria that we used to identify a sampling frame of influencer accounts can be found in Table 2.

**Table 2:** Criteria used to determine phase 1 sampling frame eligibility.

Criterion	Example
Number of social media followers	Included—greater than 10,000 followers across Tik Tok or Instagram Excluded—fewer than 10,000 followers across both platforms
Frequency of posting on Tik Tok	Included—at least 4 posts per month for past 3 months Excluded—fewer than 4 posts per month for the past 3 months
Focus on mental health	Included—at least approximately 1 out of every 3 posts in the previous six months discuss mental health Excluded—fewer than 1/3 of posts in the previous six months discuss mental health
No “red flags”	Included—creators whose content did not fit the criteria below Excluded—creators who posted harmful, misleading, or otherwise problematic content in at least one post examined
Age	Included—creators who are age 18 or older Excluded—creators who are less than 18 years old
Primary language of communication on TikTok	Included—creators whose primary language of communication on TikTok is English Excluded—creators whose primary language of communication on TikTok is a language other than English
Established account	Included—creators who have had established TikTok presence dating back at least to Jan 2022 Excluded—creators who established their TikTok presence after Feb 2022

Two research assistants were tasked with identifying eligible creators over a time period spanning from December 4, 2022 to February 2, 2023. Creators were added to our sample based on their eligibility according to the criteria listed in the table above.

After determining which accounts would be eligible for inclusion into this study, we then asked a team of two student research assistants at Harvard to search the TikTok platform for all accounts that met these criteria. As there is no TikTok “Census” of mental health content creators available to academic researchers, the research assistants employed snowball sampling techniques to generate our sampling frame.

Specifically, they took up each of the following actions to identify accounts relevant for inclusion into our study:

- Collecting word-of-mouth recommendations from members of the Harvard TH Chan School of Public Health community who study and conduct research on mental health and related subjects
- Producing lists of or coverage of top mental health content creators available on the internet by various outlets, such as The Root, Inverse, and Everyday Health
- Searching hashtags within Tik Tok including: #mentalhealth, #mentalhealthawareness, #selfcare, #selflove, #mentalillness, #therapy, #mentalhealthmatters, #mindfulness, etc.
- Browsing the “following” list of previously identified creators, since many creators in this space follow each other
- Allowing the Tik Tok recommendation algorithm to surface mental health creators

These procedures, carried out between December 2022—February 2023, allowed us to identify N = 105 accounts suitable for inclusion into the sampling frame.

We of course recognize that our efforts to identify all influencer accounts that satisfy the objectives laid out in Table 1 are necessarily imperfect. Moreover, we recognize that the criteria by which we distinguish influencer from non-influencer accounts (e.g., by employing a “reach” threshold of 10,000 followers) is open for potential definitional contestation. Our primary goal then is not (nor can it be) perfect identification of all accounts potentially suitable for inclusion in our analysis, but to create a sampling frame that is both wide in its social media reach, representative of the inclusion criteria outlined in Table 2 , and that results from a reasonably-exhaustive search of the platform for relevant influencer accounts.

### Recruitment phases #2a & 2b: assignment into study-wide treatment and control groups

After determining content creators’ eligibility for inclusion into our sampling frame (Phase 1), we next randomly assigned content creators into one of two groups. The first group is a study-wide control group, composed of content creators who were not invited to participate in our study, and not exposed to any of our experimental stimuli. Their video content serves as the primary point of analytical comparison in this study. The second is a study-wide treatment group, comprised of content creators who had the ability to opt-in to participating in our study; which we then further randomize into two different treatment groups (Phase #3; more on these procedures below).

Of course, we recognize that our sampling frame consists of only several dozen content creators, and anticipated that some proportion might opt out of participation into the study’s experimental protocols. Correspondingly, we over-assigned creators into the study-wide treatment group, such that each creator listed in the sampling frame had a 1 in 5 chance of being assigned to the study-wide control.

We next invited all content creators selected into the study-wide treatment condition to participate in our study. We began by sending an initial recruitment email on 2/9/23, which invited respondents to participate in an inaugural summit pertaining to raising awareness about mental health. Subjects were informed that they would be invited to attend 7 h long sessions as part of a virtual summit held at Harvard to discuss “cutting-edge research, emerging policy prescriptions, and critical new resources in mental health.”

Those creators who had not replied by 2/21/23 received a follow-up email re-inviting them to participate in our study’s mental health conference. Full recruitment materials can be found in the Online Appendix.

In total, 42 content creators accepted our invitation to participate in the synchronous training sessions.

Here, it is important to note that we are reluctant to consider those creators who declined our invitation to serve as a control group in our analyses as members of our control group. Although these individuals did not receive our treatment materials, they nevertheless had the *opportunity to be informed* about our study, and its objectives.

Recognizing that some level of awareness about our study’s methods could in turn influence the behavior of invited-but-untreated content creators’ behavior, we opt to avoid including these individuals in the study’s control group.

Additionally, and as noted earlier, we caveat that our experimental protocols represent an Intent to Treat (ITT) design. The fact that some creators who could have been randomized into one of our treatment groups opted out of this study implies that our analyses (and subsequent findings) are limited in scope to just those creators who were both motivated and able to participate. This is a potential limitation of our study, as highly-motivated creators may be more receptive than the population more generally to our treatment materials; i.e., because they may be comparatively more interested and willing to incorporate EBMHC into the content of the videos they produce. We therefore welcome future efforts to expand approaches like ours beyond the ITT framework, in order to understand how more heterogeneous samples of creators might respond to efforts to promote EBMHC.

### Recruitment phase #3: assignment into conference & toolkit treatment sub-groups

After allowing content creators approximately one month to opt-in to the study’s experimental protocol (i.e., to note that they planned to attend the virtual conference), we again randomly assigned respondents into one of two groups. The first group, which we refer to as the “conference & materials” group (CM) had the opportunity to both attend our conference in person *and* receive a series of digital and video materials summarizing some of the conference’s core objectives. The second group, which we refer to as the “materials only” (MO) group, was not invited to participate in the in-person conference, but was provided with all reference materials passed along to the CM group. In order to ensure sufficiently-high levels of attendance at the conference, we again over-assigned study subjects into the CM group, such that N in N + K had the opportunity to attend the conference.

The conference and supporting materials reviewed cutting-edge scientific research and emerging policy prescriptions in five areas of mental health: mental health solutions that might scale to close the global care gap (Core Theme #1), maternal mental health and how trauma can span generations (Core Theme #2), the intertwinedness of mental and physical health (Core Theme #3), the corrosive effect of bias and discrimination on mental health (Core Theme #4), and climate grief and what might be done about it (Core Theme #5). Participants were also provided practical tips for evaluating research studies and identifying and responding to visual misinformation.

Note that all materials provided to subject participants in both the CM and MO groups are available as supplementary materials (see: https://osf.io/jn8m7/).

### Content analysis

We determined whether or not each TikTok video in our dataset contained the content elements outlined in Table 1 via manual (i.e., human-coded) content analysis. To do this, We employed three research assistants (RAs) from Harvard to serve as coders for this project. Each RA was randomly assigned to code one third of the project’s N = 3465 videos—presented in a random order—with 10% (N = 351) of videos “triple assigned” to all coders for the purpose of assessing inter-coder reliability (ICR). Coders were not informed about content creators' treatment assignments prior to coding their videos.

Following a training session period — which included an iterative series of short pilot coding and feedback sessions — coders completed a short standardization assessment on a randomly selected subset of 10 videos. Coders were permitted to begin coding videos once inter-coder reliability (ICR) was sufficiently high (Gwet’s AC > 0.85) across all variables in the training set.

Note that, because many content codes are zero inflated (i.e., many more non-observations of content than detections), and because codes are non-independent (i.e., not observing content [0 codes] necessarily implies that it is not observed^1^ codes), conventional ICR statistics like Cohen’s Kappa (*k*) can produce paradoxes where *agreement* among coders is high (similar codes for content non-observation), but *reliability* is low (due to disagreements about comparatively-rarer content detection).

Gwet’s Agreement Coefficient (AC) corrects for this by relaxing independence assumptions and benchmarking reliability vs. expected *disagreement* (as opposed to agreement) statistics (Gwet 2008). In addition to the conceptual benefits of calculating ICR in situations like ours, Gwet’s AC has been employed in previous mental health content analytic research^22^.

At the project’s completion, we documented substantial levels of agreement among the coders, such that *AC* exceeded 0.90 across all EBMHC indicators. Please see Table S2 in the Online Materials for a detailed summary of *AC* values across all variables.

Note that just one of each of the “triple assigned” videos were entered into the study’s final dataset. Triple-coded videos were selected for inclusion using a random number generator contained in the duplicates suite of commands in Stata 15. 

### Analytical strategy: assessing changes in content attributable to our interventions

The primary units of analysis in models and visualizations presented throughout this piece are “creator-videos;” i.e., the content (videos) produced by each creator over time. This means that the data are structured in “long” form (multiple videos per creator). The primary outcome variables, as noted throughout this piece are dichotomous indicators of whether or not each video included the content elements featured in Table 1.

We estimate changes in content across the study’s pre- and post- intervention periods—across the study’s two treatment groups and the control group—using mixed multilevel linear probability modeling via the **mixed** suite of commands in Stata 18^23^. These models are structured hierarchically, in order to account for the possibility that some content creators may be more or less receptive to the treatments than others, by including random effect variance components.

The models then isolate the effect of change in content attributable to exposure to our study’s experimental stimuli by interacting dichotomous fixed effect indicators of treatment group assignment (MO vs. CM, with the Control serving as a reference group in both cases), with an indicator of whether or not each video was produced pre or post intervention.

For those more familiar with difference-in-difference (DiD) estimation, our approach can be thought about as being conceptually similar to conventional DiD, with the addition of random effect variance components. Additionally, despite the high levels of agreement between the project’s three coders noted above, we account for the possibility of coder-level heterogeneity in content coding by including dichotomous fixed effect indicators for each coder (with one anonymized coder serving as the reference category).

Note that, as our treatment exposure for the CM group was carried out throughout the month of April 2023, we pool all video content created in April into a “fuzzy” post-treatment period indicator (i.e., incorporating both April and May 2023). Additionally, note that we limit our analysis to only those videos determined by the project’s coders to pertain to mental health.

## Extended materials

### Methods tables

See Table 1.

### Supplementary Information

Supplementary Information.

## Data Availability

The datasets generated and/or analyzed during the current study are available in the OSF repository at the following webpage: https://osf.io/jn8m7/.
