# Supplementary material for: “Influencing the influencers:” a field experimental approach to promoting effective mental health communication on TikTok
Source: Sci Rep. 2024 Mar 11;14:5864. doi: 10.1038/s41598-024-56578-1 (PMC10928193; doi:10.1038/s41598-024-56578-1)
Supplement: Supplementary file 1 — Supplementary Information. [file 41598_2024_56578_MOESM1_ESM.docx]

**Supplemental Analyses**

|  | **Video References Any**  **Core Theme** | **Video Pertains**  **to Mental Health** |
| --- | --- | --- |
|  |  |  |
| Post Intervention | -0.08* | -0.01 |
|  | (0.04) | (0.02) |
| MO Condition | -0.03 | -0.04 |
|  | (0.07) | (0.09) |
| CM Condition | -0.02 | 0.12 |
|  | (0.06) | (0.09) |
| **MO X Post** | 0.11* | 0.06 |
|  | (0.05) | (0.03) |
| **CM X Post** | 0.04 | -0.03 |
|  | (0.05) | (0.04) |
| Coder B | 0.14* | -0.17* |
|  | (0.02) | (0.02) |
| Coder C | -0.01 | -0.25* |
|  | (0.02) | (0.02) |
| Constant | 0.27* | 0.78* |
|  | (0.05) | (0.06) |
| Variance Component: |  |  |
| Creator Random Effects | -1.93* | -1.39* |
|  | (0.13) | (0.10) |
| Variance Component: |  |  |
| Residuals | -0.89* | -0.97* |
|  | (0.01) | (0.01) |
| N | 2282 | 3434 |

**Table S1 – Main Effects of MO and CM Condition Assignment on Video Production Behavior.** *Parameter estimates from a multi-level Linear Probability Model (LPM)* *presented, with standard errors in parentheses. Video counts (N) reflect all videos from control group participants, those enrolled in the MO condition, and those who were both enrolled into the CM condition and opted into the study’s synchronous component (N = 62). Please refer to the main text for additional information about our modeling approach. Interactive coefficients referenced in text are presented in bold, on the left-hand side of the table.*

| **Content Element** | **Percent Agreement** | **Gwett’s AC Coefficient** |
| --- | --- | --- |
| Video Pertains to Mental Health | 76% | 0.69 |
| References Core Theme #1 | 96% | 0.95 |
| References Core Theme #2 | 93% | 0.92 |
| References Core Theme #3 | 94% | 0.94 |
| References Core Theme #4 | 97% | 0.97 |
| References Core Theme #5 | N/A *(insufficient N)* | N/A *(insufficient N)* |

**Table S2. Inter-Coder Reliability (ICR) Assessment.** *ICR scores are calculated across all three of the study’s coders, and presented using Gwett’s AC criterion. Please refer to the Methods section for a detailed discussion of this statistic, and its appropriateness for this study. For reference, we provide estimates of the degree to which all three raters code for the presence/absence of each dimension listed in the table (“Percent Agreement.”)*

|  | **References Core Theme 1** | **References**  **Core Theme 2** | **References Core Theme 3** | **References Core Theme 4** |
| --- | --- | --- | --- | --- |
| Post Intervention | -0.01 | -0.02 | -0.04 | -0.01 |
|  | (0.02) | (0.02) | (0.02) | (0.02) |
| MO Condition | -0.04 | 0.06 | -0.01 | -0.02 |
|  | (0.03) | (0.05) | (0.04) | (0.04) |
| MO X Post | 0.02 | 0.01 | 0.05 | 0.01 |
|  | (0.03) | (0.03) | (0.03) | (0.02) |
| CM (No Attendance) | -0.07 | 0.05 | -0.00 | -0.06 |
|  | (0.04) | (0.06) | (0.06) | (0.06) |
| CM (NA) X Post | 0.05 | -0.01 | 0.11* | 0.03 |
|  | (0.04) | (0.04) | (0.05) | (0.04) |
| CM (Attended) | -0.02 | 0.04 | 0.04 | -0.07 |
|  | (0.03) | (0.05) | (0.05) | (0.04) |
| **CM (A) X Post** | -0.02 | 0.06 | -0.08* | 0.01 |
|  | (0.03) | (0.03) | (0.04) | (0.02) |
| Coder B | 0.02 | 0.10* | 0.02 | 0.01 |
|  | (0.01) | (0.01) | (0.01) | (0.01) |
| Coder C | 0.01 | -0.06* | 0.01 | 0.00 |
|  | (0.01) | 0.10* | (0.01) | (0.01) |
| Constant | 0.08* | 0.03 | 0.10* | 0.09* |
|  | (0.02) | (0.03) | (0.03) | (0.03) |
| Variance Component: |  |  |  |  |
| Creator Random Effects | -3.41* | -2.16* | -2.42* | -2.29* |
|  | (0.26) | (0.12) | (0.13) | (0.11) |
| Variance Component: |  |  |  |  |
| Residuals | -1.46* | -1.38* | -1.27* | -1.60* |
|  | (0.01) | (0.01) | (0.01) | (0.01) |
| **N** | 2282 | 2282 | 2282 | 2282 |
|  |  |  |  |  |

**Table S3 – Main Effects of CM Session Attendance on Video Production Behavior.** *Parameter estimates from a multi-level Linear Probability Models (LPM)* *presented, with standard errors in parentheses. Video counts (N) reflect all videos from control group participants, those enrolled in the MO condition, and those who were both enrolled into the CM condition and opted into the study’s synchronous component (N = 62). Please refer to the main text for additional information about our modeling approach.*

|  | **Video References**  **Any Core Theme** | **Video Pertains to Mental Health** |
| --- | --- | --- |
| Post Intervention | -0.00 | 0.01 |
|  | (0.02) | (0.03) |
| MO Condition | 0.06 | -0.15 |
|  | (0.07) | (0.10) |
| CM Condition | 0.02 | -0.01 |
|  | (0.07) | (0.12) |
| MO X Post | 0.03 | 0.01 |
|  | (0.03) | (0.04) |
| CM X Post | 0.09* | -0.07 |
|  | (0.04) | (0.06) |
| Large Following (>2M) | 0.23* | -0.21 |
|  | (0.09) | (0.12) |
| Post X Large Following | -0.14* | -0.04 |
|  | (0.06) | (0.04) |
| Coder B | 0.14* | -0.17* |
|  | (0.02) | (0.02) |
| Coder C | -0.01 | -0.25* |
|  | (0.04) | (0.03) |
| MO X Large Following | -0.08 | 0.21 |
|  | (0.15) | (0.17) |
| CM X Large Following | -0.11 | 0.28 |
|  | (0.12) | (0.16) |
| **MO X Post X Large Following** | 0.16* | 0.18* |
|  | (0.07) | (0.07) |
| **CM X Post X Large Following** | -0.05 | 0.07 |
|  | (0.08) | (0.07) |
| Coder B | 0.14* | -0.17* |
|  | (0.02) | (0.02) |
| Coder C | -0.01 | -0.25* |
|  | (0.04) | (0.03) |
| Constant | 0.15* | 0.89* |
|  | (0.07) | (0.08) |
| Variance Component: |  |  |
| Creator Random Effects | -2.02* | -1.43* |
|  | (0.13) | (0.09) |
| Variance Component: |  |  |
| Residuals | -0.89* | -0.97* |
|  | (0.05) | (0.04) |
| N | 2282 | 3434 |

**Table S4 – Moderated Effects of MO and CM Intervention Assignment on Video Production Behavior.** *Parameter estimates from a multi-level Linear Probability Models (LPM)* *presented, with standard errors in parentheses. Note that, given the comparatively complex structure of these three-way interactive models, we reduce the possibility of committing Type II error by estimating robust standard errors. Please refer to the main text for additional information about our modeling approach. Video counts (N) reflect all videos from control group participants, those enrolled in the MO condition, and those who were both enrolled into the CM condition and opted into the study’s synchronous component (N = 62).*

**Supplemental Recruitment Materials**

**Figure S1. Initial Phase 2 Subject Recruitment Email Sent on 2/9/23**

*Note. Please see Methods for a description of the Phase 2 randomization protocols.*

| Hi [name]    The Harvard School of Public Health will convene an exclusive group of 25 leading mental-health creators for a free, online summit in April. Will you join us and share your expertise about creating mental health content?    We love what you are doing to raise awareness about mental health and would be honored to have you **apply to join this inaugural summit**.    · During 7 hourlong sessions held throughout the month, this cohort of creators will engage with experts and clinicians at Harvard and beyond to talk about cutting-edge research, emerging policy prescriptions, and critical new resources in mental health.  · Expect a highly interactive summit at which you’ll learn, share, connect­—and shape the future of mental health communication.    You can find more information about what we have planned **here**. The deadline for applications is **March 1.**    *Plus: Creators who attend the virtual summit will also have the chance to reconvene in real life this summer on the Harvard* *campus, at our expense, to help shape the future of mental health communication.*    We hope you’ll join us for this exciting Harvard program. Apply **here**. |
| --- |

**Figure S2. Follow-Up Phase 2 Subject Recruitment Email Sent on 2/21/23**

*Note. Please see Methods for a description of the Phase 2 randomization protocols.*

| Hi [name]    The deadline for Harvard Chan School’s Creator Summit on Mental Health is coming up next week, and slots are filing up fast! We’d be thrilled to see you apply to join this exclusive cohort of 25 creators shaping the future of mental health communication.    Let us know if you’d like to jump on a call to talk more about this opportunity. |
| --- |

**Figure S3. Follow-Up Phase 2 Subject Recruitment Email Sent on 2/28/23**

*Note. Please see Methods for a description of the Phase 2 randomization protocols.*

| Tomorrow is the deadline for the Harvard Chan School’s Creator Summit on Mental Health on Mental Health in April.    Slots in this free summit for top mental health creators are filing up fast!    Here are some of the reasons why other top creators have told us they applied:  · To learn about the latest research on the toxic effects of discrimination, the mind-body connection, and more  · To have the opportunity to moderate Q&As with Harvard faculty  · To visit Harvard this summer to interact with faculty, students, and other creators, at Harvard’s expense  · To network with and learn from other top mental health creators  · To shape the future of mental health communication and forge new academic-creator partnerships    Please explore our April agenda: <https://hsph.me/creators-agenda>  And apply today! <https://hsph.me/creators-apply>    Note: Creators are encouraged to attend every session but if you have a conflict, we’ll provide exclusive briefings to help you catch up on anything you missed.  If you have any questions, please reach out! |
| --- |
